# Long-term effects of adding biochar to soils on organic matter content, persistent carbon storage, and moisture content in Karagwe, Tanzania

**DOI:** 10.1038/s41598-024-83372-w

**Published:** 2024-12-19

**Authors:** Baraka Ernest, Pius Z. Yanda, Anders Hansson, Mathias Fridahl

**Affiliations:** 1 Department of Medical Botany, Plant Breeding, and Agronomy, Muhimbili University of Health and Allied Sciences, P.O. Box 65001, Dar es Salaam, Tanzania; 2 Institute of Resource Assessment, University of Dar es Salaam, P.O. Box 35097, Dar es Salaam, Tanzania; 3 Department of Thematic Studies: Environmental Change, Centre for Climate Science and Policy Research (CSPR), Linköping University, SE-58183 Linköping, Sweden

**Keywords:** Climate-change ecology, Climate change, Climate-change mitigation, Climate-change ecology, Ecosystem ecology, Restoration ecology

## Abstract

Soils require the application of biochar to improve degradation. The objective of this study was to evaluate the long-term effects of a field experiment on soil organic matter (SOM), soil organic carbon (SOC), and soil moisture content in Karagwe, Tanzania. Seven years ago, a field experiment was conducted using a Latin rectangle design with four replications. The treatments included carbonization and sanitation (CaSa) and carbonization and standard compost (CaSt), which were compared to control Andosols (CoA). A total of 96 soil samples were collected to determine SOM, SOC, and soil moisture content. The data were analyzed using one-way analysis of variance. The results showed that soil samples from the CaSa-treated soil had an increase in SOM content of 17.3%, an increase in stored SOC content of 10.0%, and an increase in soil moisture content of 6.3%. Compared with those in CoA, the CaSt-treated soil showed increases in SOM, SOC, and soil moisture of 14.4%, 8.4%, and 4.0%, respectively. Therefore, all treatments improved soil properties, with CaSa proving more effective in enhancing SOM, SOC, and soil moisture content compared to CaSt and CoA. In conclusion, CaSa is recommended for its sustainable ability to enhance Karagwean soil over time.

## Introduction

The soil is the most significant component on Earth’s surface and encompasses a complex ecosystem that includes microorganisms, inorganic particles and soil organic matter (SOM)^1–3^. SOM consists of decomposed plant remnants, microbes, and the products of microbial breakdown^4–6^. SOM is essential for maintaining soil quality and sustainability over time^7,8^. Soil organic carbon (SOC) refers to the amount of carbon stored in the soil after the organic matter content has partially decomposed^9^. Approximately 1500 petagrams of organic carbon are held in the soil, making soil the largest carbon resource in the terrestrial biosphere^10–12^. SOC is essential for sustaining ecological stabilization because it promotes the fixation of carbon dioxide (CO_2_), supports the global carbon cycle, and enhances soil fertility^13–16^. Soil moisture refers to the water content in the active layer of the soil and is usually measured at a depth of 1–2 m^17^. Adequate soil moisture is necessary for regulating plant growth^18^, soil temperature^19^, microbial activity^20,21^, and nutrient transportation^22^ and ensuring agricultural water security^23^.

However, approximately 25% of the world’s fertile soil resources have experienced a significant decline in quality due to human activity^24^. For instance, inadequate soil management practices in sub-Saharan Africa (SSA) have resulted in the degradation of approximately 25% of fertile soils^25^. In Tanzania, continuous plowing poses a threat to Karagwean soil, as farmers fail to replenish the soil with sufficient organic amendments^26–30^. Consequently, this leads to erosion, causing the deterioration of the SOM, a reduction in the soil’s ability to retain SOC, and the loss of soil moisture^31–33^. These practices have threatened more than 65% of people in SSA who depend on agriculture for their livelihood^34^. Therefore, to achieve sustainable soil management, it is crucial to implement the use of biochar as a long-term strategy that focuses on increasing SOM, SOC, and soil moisture content^35,36^.

Through pyrolysis, biomass is converted into biochar, a stable carbon-rich product at high temperatures between 300 and 900 °C in the absence of oxygen or in a limited oxygen environment^37–39^. Studies suggest that biochar is porous and has a high surface area and significant negative charges, which improve soil properties^40,41^. Biochar is valuable as an organic amendment because of its ability to change SOM content and retain soil moisture^42,43^, increase soil carbon storage and capture carbon from the atmosphere^44^. The long-lasting recalcitrant qualities of biochar help maintain carbon stocks and slow the degradation of SOM^45,46^. However, the adoption of biochar in the Karagwe district is limited by an unstable supply of feedstocks, inadequate knowledge about the impacts of biochar on soils, restricted access to financial resources, and limited production techniques^47^.

In response to these challenges, the Carbonization and Sanitation (CaSa) project and the Efficient Cooking in Tanzania (EfCoiTa) project were launched in Karagwe in 2014. These projects aim to promote the environmental benefits of sanitation and thermal cooking technology while improving soil quality sustainably by utilizing locally available biomass^48^. Along with producing powdered charcoal, known as “biochar,” the projects utilized upgraded top-lit updraft (TLUD) cook stoves for cooking and sanitation purposes. The produced biochar was subsequently used as an organic amendment to restore degraded soil^48,49^. Therefore, the use of biochar is a long-term, viable strategy for sustainably improving degraded soil^50–52^.

Most studies have conducted short-term experiments (within a single planting season) to evaluate the effects of biochar on soil properties^53–55^. For instance, in Heilongjiang Province, China, Li et al.^56^ conducted a short-term experiment and found that applying biochar had no effect on the overall pattern of soil moisture content. In Tanzania, Krause et al.^49^ conducted a short-term field experiment in Karagwe on the influence of biochar on soil fertility, carbon storage, and water content. However, no noticeable change in soil carbon content was observed during the first planting season following the application of biochar^49^. Therefore, these short-term field experiments are often criticized for their failure to reveal changes in SOM, carbon storage, and soil moisture content^49^. To uncover the long-term effects of biochar, a study conducted in China by Cong et al.^57^ demonstrated that a one-time application of biochar in the field over seven years had long-lasting positive impacts on improving soil quality. Despite the existence of some field studies on the long-term impacts of biochar, there is a global scarcity of long-term studies that consider various regions and soil types^57^. Therefore, there are limited long-term studies evaluating how biochar addition to soil affects SOM, carbon storage, and moisture content for several years in Karagwe^49,58,59^.

Due to the limited number of long-term studies on the effect of biochar on soil quality in degraded areas^60,61^, it is essential to conduct long-term field experiments to fully comprehend the impact of biochar on enhancing degraded soil quality^62^. However, studies have shown that adding biochar to a field experiment once can improve soil properties over several years^57,63^. To address the existing information gap, this study aimed to evaluate the long-term effect of one-time biochar incorporation in Karagwean soil on the organic matter content, persistent carbon storage, and moisture content after seven years. The one-time addition of biochar may have long-term impacts due to its stability, allowing it to remain in the soil for millennia^57,64^. Therefore, we hypothesize that adding biochar to Karagwean soil once for seven years may increase the SOM content, increase carbon storage, and increase the moisture content.

## Materials and methods

### Preparation of biochar

The TLUD cook stove, located in Karagwe, Tanzania, was used as part of the CaSa and EfCoiTa project to produce the biochar. The biochar used in this study was created through carbonization and sanitation (CaSa), which involved combining eucalyptus sawdust with pasteurized human excreta (a mixture of urine and feces). Another type of biochar, known as carbonization and standard compost (CaSt), was also used in this study. CaSt biochar is made from eucalyptus, a blend of grasses, ash, bean straw, and banana peels. According to the World Health Organization^65^, urine can be safely used as an organic fertilizer in agriculture over a long period of time. However, feces contain high levels of bacteria, protozoan cysts, viruses, and worm eggs, which are necessary to treat before use^65,66^.

Innovative approaches to sanitation, such as urine-diverting dry toilets (UDD) and TLUD cook stove pasteurization, allow us to view human excrement as a valuable resource rather than mere waste^59^. The feedstocks used in this study were pyrolyzed in a TLUD cook stove at a high temperature of 500 °C^48^. Pasteurized human excrement is an effective means of recycling micronutrients, nitrogen (N), phosphorus (P), and potassium (K) in plants^49^. Furthermore, the use of biochar produced in home gardens not only provides agronomic benefits but also helps reduce carbon emissions^67^. The physicochemical properties of the biochar were analyzed in a previous article by Krause et al.^48^.

### Study site description

**Fig. 1:**
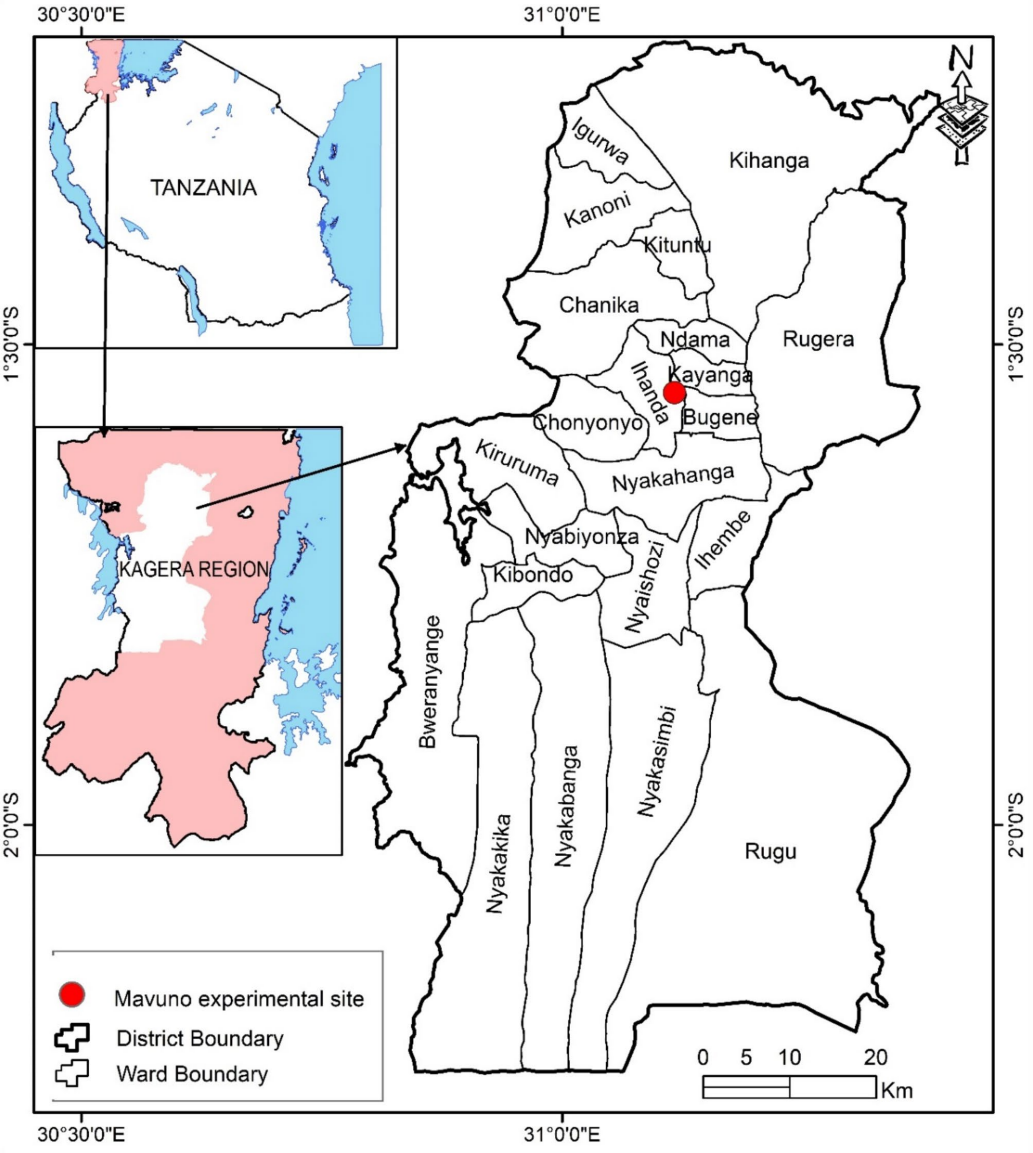
Map showing the location of the Mavuno experimental site in Karagwe, Tanzania. The map was generated using ArcGIS 10.8 software. Source: University of Dar es Salaam, Institute of Resource Assessment, Geographic Information System (GIS) Laboratory, 2024 (https://www.udsm.ac.tz/web/index.php/institutes/ira).

The Mavuno Organization, located in Karagwe, Tanzania, operates at the Mavuno experimental site where this assessment took place. The coordinates of the site are 1° 34’ 22.4” South and 31° 03’ 28.6” East^49^ (Fig. 1). Due to the semiarid tropical savanna environment in the area, most crops can be harvested twice a year^68^. Rainfall occurs in the region twice a year, from September to November and from March to May^69,70^. According to the United Republic of Tanzania^70^, the average annual temperature ranges from 20 to 28 °C, and the soil type is classified as an Andosol^71^. The following are the fundamental characteristics of this soil classification, which were sampled at a depth of 0 to 30 cm: pH of 3.8, cation exchange capacity of 16.7 cmolkg^− 1^, total nitrogen of 0.3%, available phosphorus of 0.7 mgkg^− 1^, available potassium of 244.7 mgkg^− 1^, bulk density of 0.9 kgdm^− 3^, total SOC of 3.5%, and SOM of 6.0%.

### Experimental design and soil sampling

**Fig. 2:**
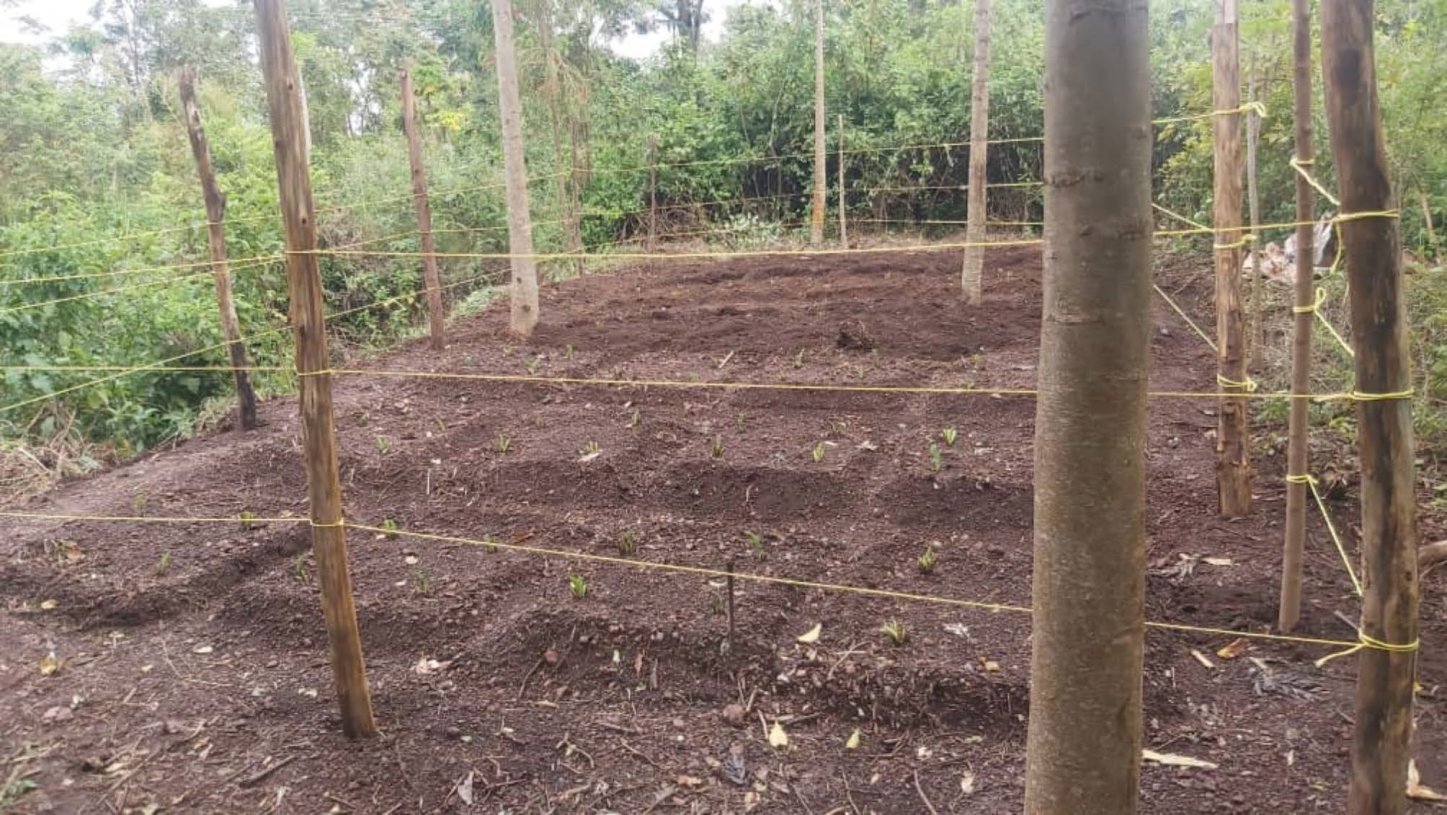
An experimental field to showcase various blocks and plots.

The short-term experimental study of March 2014 consisted of a series of 3 × 3-meter plots arranged in the shape of a Latin rectangle to reduce carry-over effects throughout the experiment, with five columns and five rows (Fig. 2). Each plot was separated from the others by a 0.5-meter-deep trench^49^. Similar to previous studies, there were five replications for each row and column, and four different treatments were applied.

In the present study, which focused on the long-term effects of biochar on SOC, SOM, and soil moisture content, both replications and treatment counts were modified within a comparable experimental field. However, the long-term addition of biochar to the soil gradually diminishes its effects due to soil leaching, which requires repeated applications to maintain its reparative properties^72^. Biochar can persist in the soil for thousands of years due to its inherent stability, allowing a one-time application to have long-lasting impacts on soil function^64^. This enables us to conduct and evaluate the long-term effects of a one-time biochar application on improving degraded soil quality over a seven-year period. Therefore, we evaluated the experimental field using a set of 3 × 3-meter plots arranged in the shape of a Latin rectangle with three columns and four rows. Latin rectangles that are counterbalanced for an odd number of treatments with reduced columns and rows are referred to as Youden rectangles^73^. The treatments included the following: (1) untreated Andosol (CoA) serving as the control group; (2) the application of CaSt at 15.0 dm^3^ m^-2^; and (3) the application of 8.3 dm^3^ m^-2^ of CaSa^49^. The effects of the treatments were evaluated with four replications.

In August 2021, soils were collected from the experimental field that was established in March 2014 (seven years ago). For each replicate, eight subsamples were collected, and any litter, grasses, or vegetative remnants were removed following the methods of Fernández-Ugalde et al.^74^. Collecting multiple soil samples for each replicate allows for the estimation of an error term that considers various characteristics of the field experiment by capturing the variability from each replicate^75^. An auger corer was used to collect subsamples from a depth of 0 to 30 cm. This sampling depth was chosen because it is the depth at which most soil changes occur, considering the impact of soil management practices on agricultural systems and land-use types^76^. A total of 96 subsamples (number of treatments × number of replicates × number of subsamples = 3 × 4 × 8) were collected, and each subsample weighed 500 g. These samples were then sealed in plastic bags with labels and sent to the soil laboratory at Sokoine University of Agriculture for analysis of SOC, SOM, and soil moisture content.

### Laboratory analysis of SOM, SOC, and soil moisture content

Upon arrival at the laboratory, the soil samples were weighed and dried in an oven at 105 °C for 48 h until a constant weight was achieved, following the procedure outlined by Sparks et al.^77^. Immediately after cooling, the dry soil samples were analyzed using the gravimetric method to estimate the moisture content. The remaining soil samples were left to air dry at room temperature. Once air-dried, the soil samples were sieved using a 2 mm diameter sieve to retain only particles smaller than or equal to 2 mm in size.

The SOC content was determined using the Walkley-Black wet oxidation method. Organic carbon was estimated from soil aggregates obtained by sieving soil samples. Each air-dried soil sample consisted of 5 g of 2 mm soil sieved using a 0.25 mm sieve. The purpose of sieving soil samples with a 0.25 mm sieve was to increase the homogeneity of the soil particles, improve the interaction between the soil and added reagents, and reduce the variability in the estimated carbon values. After passing through a 0.25 mm sieve, a 0.5 g soil sample was placed in a flask. Then, 20 mL of 5% potassium dichromate (K_2_Cr_2_O_7_) and 10 mL of 95% concentrated sulfuric acid (H_2_SO_4_) were added to the flask as described by Walkley and Black^78^. Afterwards, the flask was placed on a heating plate and heated until the solution inside the flask began to boil at a temperature of 150 °C. After cooling, 10 mL of 95% phosphoric acid (H_3_PO_4_) and 100 mL of distilled water were added. Five drops of the ferroin indicator were added before titration. Then, 0.5 M ferrous ammonium sulfate ((NH_4_)_2_Fe(SO_4_)_2_.6H_2_O) was added, and the titer of the compound was recorded for the estimation of oxidizable soil organic carbon (OSOC)^79^. According to Estefan et al.^80^, to estimate the SOC, the amount of OSOC was multiplied by a factor of 1.3, derived from 100/77. Aregahegn^81^ explained that 77% (w/w) of the active forms of SOC are oxidized by K_2_Cr_2_O_7_ at a temperature of approximately 120 °C, facilitated by the heat of dilution reaction triggered by the addition of H_2_SO_4_. According to Estefan et al.^80^, the following equation was used to estimate the SOC:

$$\text{SOC} \left(\%\right)=\text{OSOC} \left(\%\right) \times 1.3$$

To estimate the SOM, Braisdell et al.^82^ stated that the SOC is converted using the Van Bemmelen correction factor of 1.7, derived from 100/58. This conversion assumes that SOM comprises 58% (w/w) of the SOC and is provided in the following equation^80^:

$$\text{SOM} \left({\%}\right)=\text{SOC} \left({\%}\right) \times 1.7$$

### Statistical analysis

Statistical analysis was conducted using InStat 3 software. The Tukey–Kramer post hoc test was used to determine differences between treatment means, and all the statistical tests were considered significant at *p* ≤ .05. One-way analysis of variance (ANOVA) was used to identify significant differences in SOM, SOC, and soil moisture content among the treatments.

## Results

### Effect of biochar on the SOM content

**Fig. 3:**
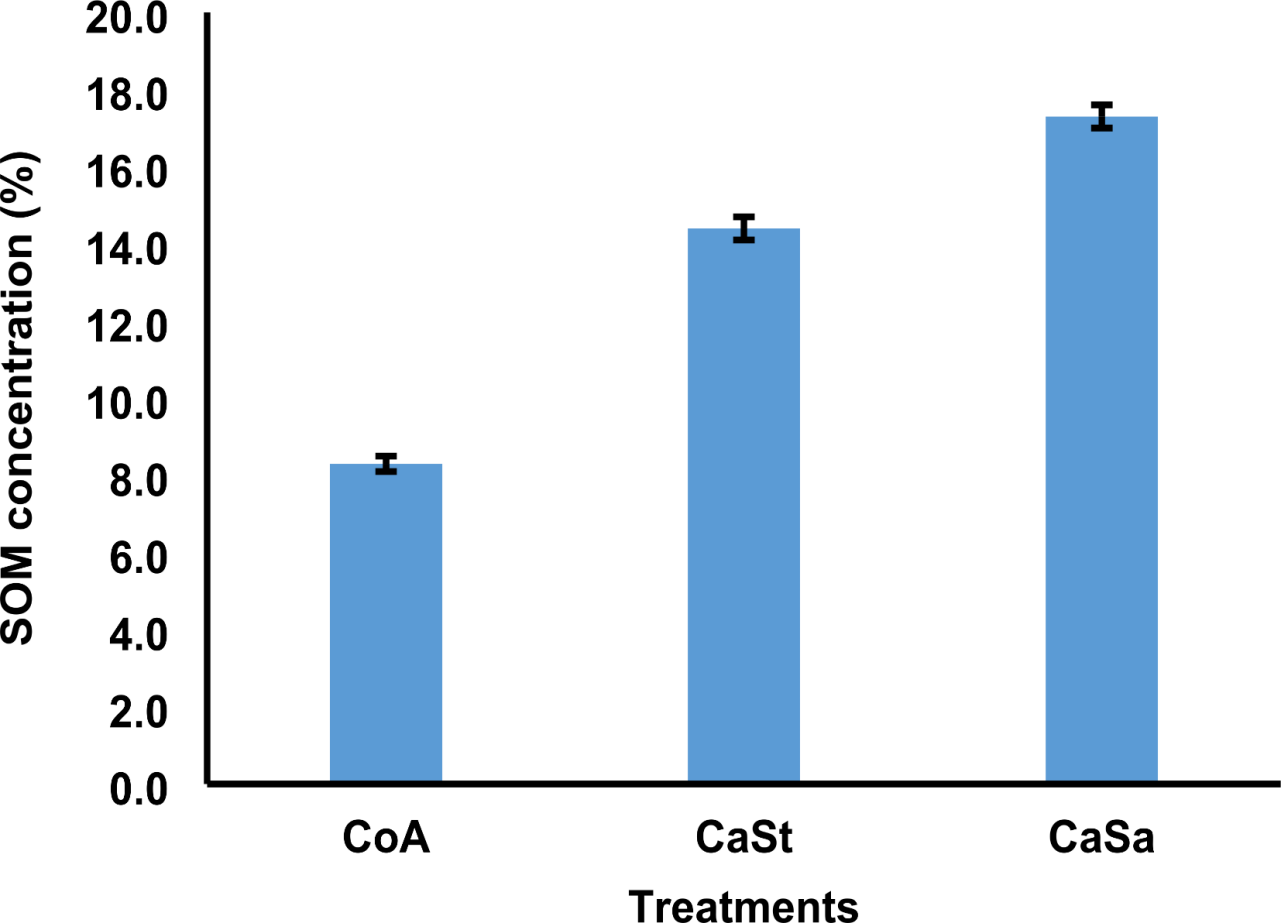
The percentage concentration of SOM. CoA represents the control andosol, CaSt represents carbonization and standard compost, and CaSa represents carbonization and sanitation. Error bars represent the standard error for each treatment mean.

Following the application of biochar, we observed that the plots treated with CaSa (17.3%) and CaSt (14.4%) had the highest concentration of SOM throughout the study. Figure 3 illustrates that the SOM content was lowest (8.3%) in the CoA plots. To further investigate these differences, we conducted pairwise comparisons and utilized the Tukey–Kramer post hoc test. The results clearly indicated significant disparities (*p* < .001) between CoA and CaSt, CoA and CaSa, and CaSt and CaSa (Table 1).

### Effect of biochar on the SOC content

**Fig. 4:**
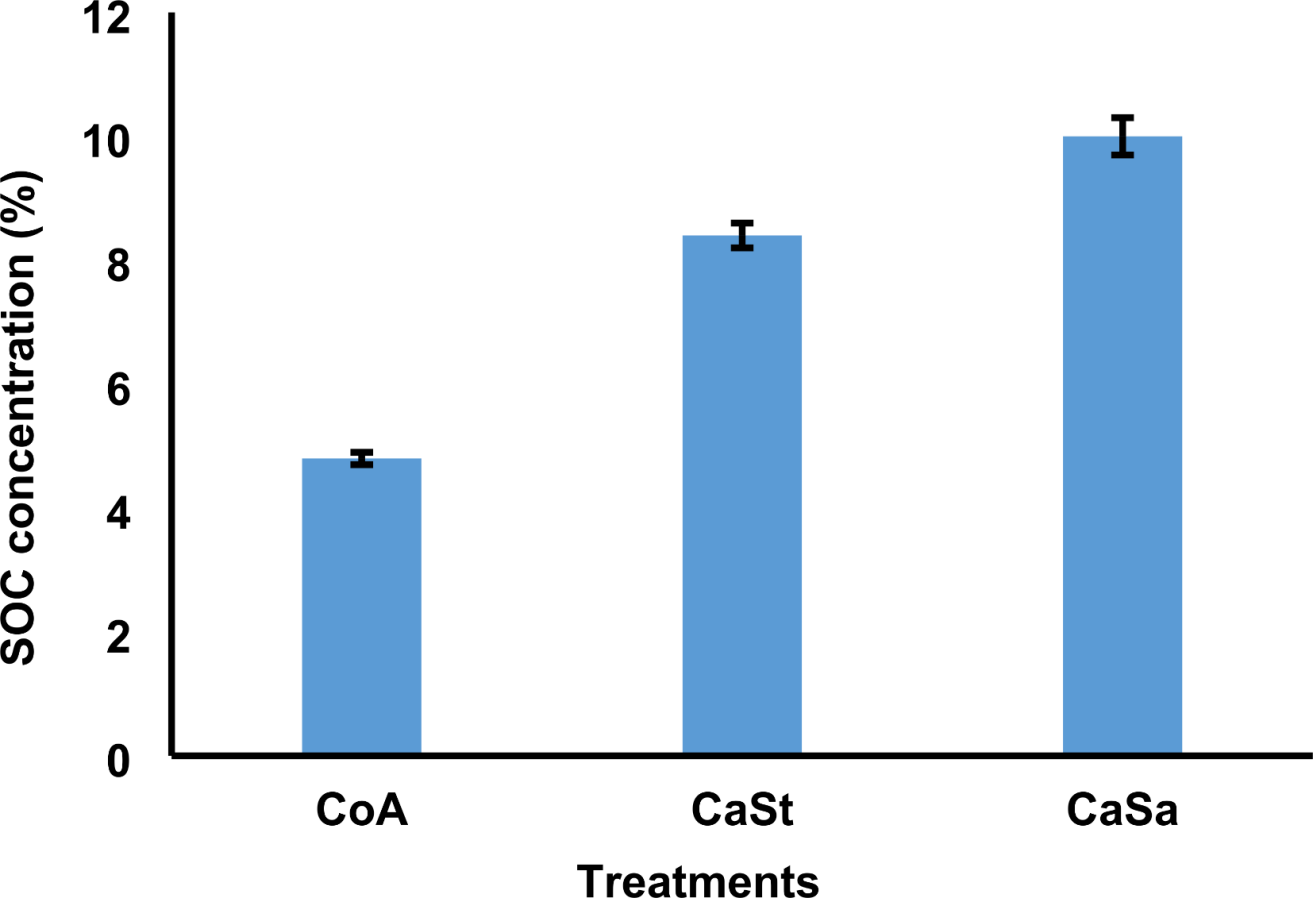
Shows the percentage concentration of SOC. CoA represents the control Andosol, CaSt represents carbonization and standard compost, and CaSa represents carbonization and sanitation. The error bars represent the standard error for each treatment mean.

The plots treated with CaSa had the highest and most statistically significant difference in SOC content, at 10.0%. This was followed by plots treated with CaSt, which had an SOC content of 8.4%. The plots treated with CoA had the lowest SOC content, at 4.8% (Fig. 4). The data indicate that there were significant differences between CoA and CaSt, as well as between CoA and CaSa, with *p* values less than 0.001. There were also significant differences between CaSt and CaSa, with a *p* value less than 0.001, as shown by Tukey Kramer’s post hoc test (Table 1).

### Effect of biochar on soil moisture content

**Fig. 5:**
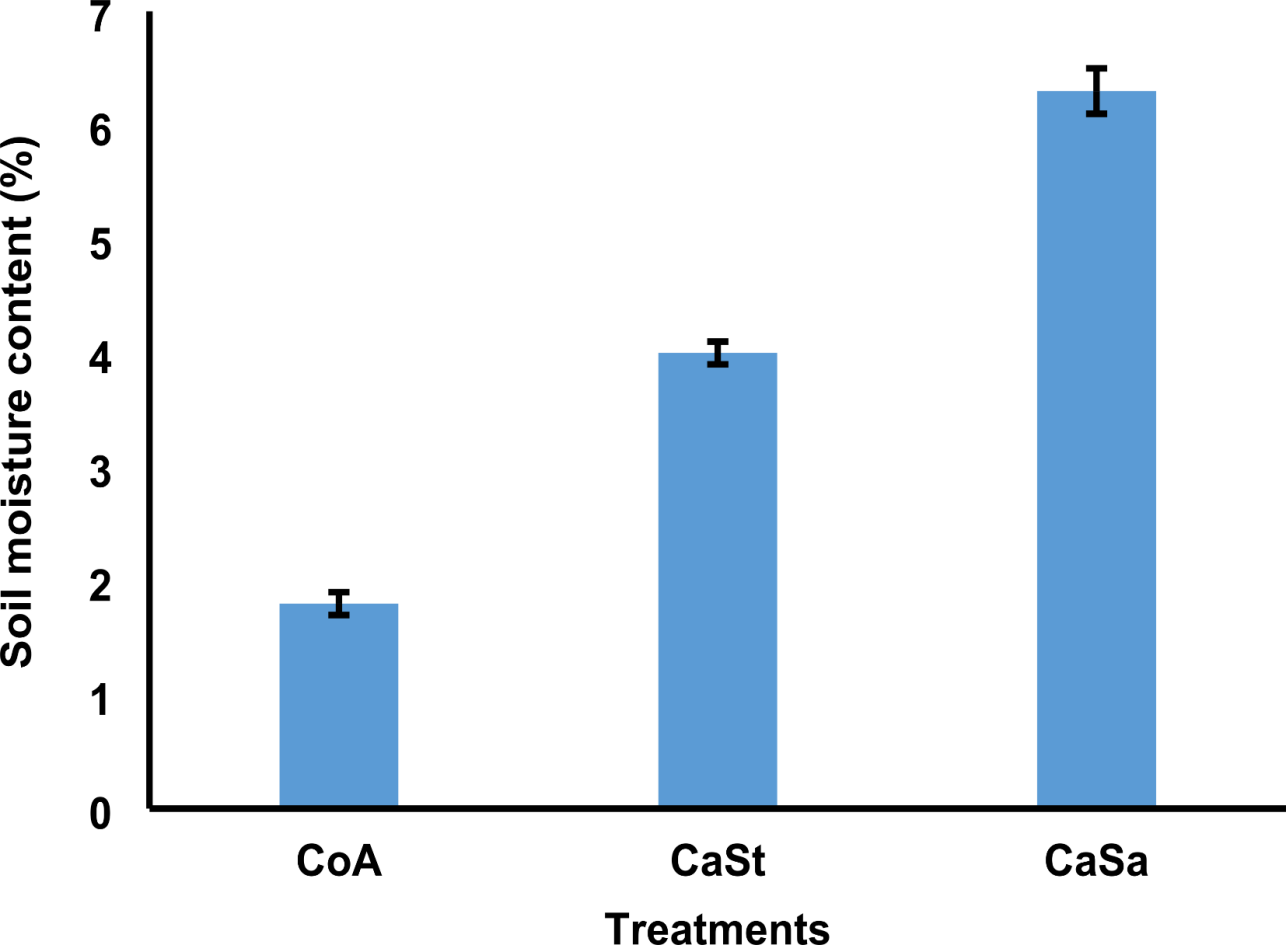
Shows percentage concentration of the soil moisture content. CoA represents the control andosol, CaSt represents carbonization and standard compost, and CaSa represents carbonization and sanitation. Error bars represent the standard error for each treatment mean.

According to the data, the soil moisture content was higher in the plots treated with CaSa (6.3%) than in the plots treated with CaSt (4.0%). Conversely, the soil moisture content was comparatively lower in the CoA plots (1.8%) (Fig. 5). When pairwise comparisons were performed using Tukey Kramer’s post hoc test (Table 1), significant variations in soil moisture content were observed between CoA and CaSt, CoA and CaSa, and CaSt and CaSa, all with *p* < .001.

**Table 1:** Mean comparisons between treatments were performed using Tukey–Kramer post hoc tests.

Pairwise comparison of treatments	A significant level of selected soil properties		
	SOM	SOC	Soil moisture
CoA compared to CaSt	< 0.001	< 0.001	< 0.001
CoA compared to CaSa	< 0.001	< 0.001	< 0.001
CaSt compared to CaSa	< 0.001	< 0.001	< 0.001

CoA represents the control andosol, CaSt represents carbonization and standard compost, CaSa represents carbonization and sanitation, SOC represents soil organic carbon, and SOM represents soil organic matter.

## Discussion

Long-term application of biochar significantly increased the amount of SOM in soils that had been treated with biochar. The plots treated with biochar had higher concentrations of SOM (Fig. 3), possibly due to the presence of organic matter in the biochar. These findings are consistent with previous studies by Khan et al.^83^ and Zhang et al.^84^, who also reported a significant increase in the SOM concentration following biochar application. According to reports by Gong et al.^85^ and Liu et al.^86^, the addition of biochar consistently leads to an increase in SOM due to its organic matter content.

According to our findings, adding biochar to soil improves the long-term preservation of SOM. Studies by Rombolà et al.^87^ and Smebye et al.^88^ have shown that the stability of biochar prevents organic matter from draining out of the soil and protects it from physical degradation. Therefore, biochar can effectively stabilize and protect SOM for many years^46^. Furthermore, our findings support the feasibility of using biochar to improve organic matter content in degraded soils for many years. Maintaining SOM at a threshold level is crucial for the balanced functioning of agroecosystems and for environmental functions^89^. It also plays a vital role in the global carbon cycle^90^.

The results of our study indicate that the addition of biochar significantly increased the amount of organic carbon in the soil. Previous research has shown that applying biochar as an organic amendment can effectively increase SOC levels^91^. Studies by Yang et al.^92^ and Lehmann et al.^93^ suggest that biochar can aid in carbon storage and expand the carbon pool. Figure 4 illustrates the differences in SOC resulting from the organic carbon component in the biochar. These findings support previous research by Nogués et al.^94^ and Rombolà et al.^87^, which indicated that biochar needs to be resistant to degradation over time to effectively store carbon in the soil. The stable structure of biochar prevents the oxidation of organic carbon and increases its resistance to microbial degradation^95,96^.

According to several studies, biochar can remain in the soil for a long time and can store carbon in a resistant form for hundreds or even thousands of years^38,46^. These findings suggest that biochar is an effective soil amendment for storing carbon and preventing its immediate release into the atmosphere. These results support the findings of Lehmann et al.^93^ and Oliveira et al.^97^, who observed that biochar, due to its high stability, can persist in the soil and benefit soil carbon sequestration. Therefore, these findings agree with the fact that biochar has the potential to provide a substantial and long-lasting carbon reservoir. Other studies have shown that SOC significantly contributes to global climate change and plays a crucial role in the global carbon balance^87,98^. These findings support the notion that biochar can serve as a valuable organic supplement for reducing carbon emissions^99,100^.

According to the results, the application of biochar significantly increased the moisture content of the soil (Fig. 5). These findings are consistent with previous studies by Edeh et al.^101^ and Murtaza et al.^102^, who showed that biochar can improve various soil physiochemical parameters, including water infiltration, water holding capacity, and saturated hydraulic conductivity, to support cultivation. Similarly, researchers Liao & Thomas^103^ and Novak et al.^104^ reported a significant increase in soil moisture content with the addition of biochar. The specific characteristics of the biochar and the application rate may have influenced the notable increase in soil moisture observed in this study.

Further support for the positive impact of biochar on soil moisture can be found in the study conducted by Asai et al.^105^, which demonstrated that the application of biochar improved the soil moisture content. This improvement is primarily attributed to the porosity and surface area of the biochar. Additionally, research by Novak et al.^104^ and Chen et al.^106^ have shown that the presence of biochar in a soil system can enhance moisture storage by altering the distribution of soil pore sizes, leading to improvements in soil aggregation. However, it is important to note that the impact of biochar on soil texture also plays a significant role in determining the soil moisture content^107^. Consequently, the application of biochar offers the advantage of increased stability, which can prolong the beneficial effects of the amendment and provide soil moisture for a longer period of time.

## Conclusion

The application of biochar as an organic amendment was found to affect soil moisture, soil organic matter (SOM), and soil organic carbon (SOC) in this study. However, the amount of SOM and SOC stored in the soil significantly increased with the application of carbonization and sanitation (CaSa). CaSa can act as a carbon pool, helping to offset carbon emissions and maintain carbon in the soil for a longer period of time. Biochar can improve soil health for cultivation, as evidenced by the increase in SOM and soil moisture content. In contrast, the control Andosol (CoA) had minimal long-term effects on the SOM concentration, SOC storage, and soil moisture content. Due to its ability to gradually increase SOM, SOC, and soil moisture, CaSa biochar is recommended for use as an organic amendment in the agricultural land of the Karagwean dominated by Andosols. However, additional research is needed to better understand how soil nutrients, physical characteristics, and heavy metal loads change over time in soils treated with biochar in similar settings.

## Data Availability

The authors certify that all the data supporting the information contained in this study are provided within the manuscript. For legitimate inquiries, the authors will provide the relevant data. For further information on the data used in this study, please contact Baraka Ernest (barakaernest690@gmail.com).
